# The typical red cell makes too little vacuole material for coalescence to build the large vacuoles of pocked cells

**Published:** 2026-10-20

**Authors:** Zhaojie Chai, Jianlu Zheng, He Li, Ming Dao, George Em Karniadakis

## Abstract

Red blood cells carrying intracellular vacuoles, called pocked cells, are a sensitive marker of splenic function: about 0.5% of cells in healthy people, 20 to 70% after splenectomy. Vacuoles too small to see are common, and visible ones are thought to grow when small ones merge. We tested that idea with a particle model that resolves the cell's membrane, its contents and every vacuole, and with an inventory of the vacuole volume a cell can make. In tube, shear and vascular flows, a cell's vacuoles met within a second, and, allowed to fuse, ten merged into one to five. Meeting is not what limits growth; material is. At the rate measured after splenectomy, all the vacuoles an average cell makes in its 120-day life fuse into one body 0.6 $μ$m across, the size healthy cells carry, but 16 times smaller in volume than the 1.5-$μ$m bodies of patients without a spleen. Taking vacuole size as set at formation, a population model in which vacuoles are made mainly in young cells, and far faster in a small minority that alone makes material for a large one, accounts for the published counts, sizes and age profiles and, on first-hand measurements, favours a splenic clearance that rises several-fold as cells age; letting that minority's vacuoles fuse fits nearly as well. At a model splenic slit, the cell's spare surface decided passage, and a vacuole mattered only when it led. A slit experiment would test whether brief encounters fuse vacuoles.

